# Carboxylated Mesoporous Carbon Nanoparticles as Bicalutamide Carriers with Improved Biopharmaceutical and Chemo-Photothermal Characteristics

**DOI:** 10.3390/molecules30153055

**Published:** 2025-07-22

**Authors:** Teodora Popova, Borislav Tzankov, Marta Slavkova, Yordan Yordanov, Denitsa Stefanova, Virginia Tzankova, Diana Tzankova, Ivanka Spassova, Daniela Kovacheva, Christina Voycheva

**Affiliations:** 1 Department of Pharmaceutical Technology and Biopharmaceutics, Faculty of Pharmacy, Medical University of Sofia, 1000 Sofia, Bulgaria; btzankov@pharmfac.mu-sofia.bg (B.T.); mslavkova@pharmfac.mu-sofia.bg (M.S.); 2 Department of Pharmacology, Pharmacotherapy and Toxicology, Faculty of Pharmacy, Medical University of Sofia, 1000 Sofia, Bulgaria; yyordanov@pharmfac.mu-sofia.bg (Y.Y.); denitsa.stefanova@pharmfac.mu-sofia.bg (D.S.); vtzankova@pharmfac.mu-sofia.bg (V.T.); 3 Department of Pharmaceutical Chemistry, Faculty of Pharmacy, Medical University of Sofia, 1000 Sofia, Bulgaria; d.tsankova@pharmfac.mu-sofia.bg; 4 Institute of General and Inorganic Chemistry, Bulgarian Academy of Sciences, 1000 Sofia, Bulgaria; ispasova@svr.igic.bas.bg (I.S.); didka@svr.igic.bas.bg (D.K.)

**Keywords:** mesoporous carbon nanoparticles, drug delivery, functionalization, bicalutamide, encapsulation, prostate cancer, photothermal therapy

## Abstract

Prostate cancer is a serious, life-threatening condition among men, usually requiring long-term chemotherapy. Due to its high efficacy, bicalutamide, a non-steroidal anti-androgen, has widespread use. However, its poor water solubility, low oral bioavailability, and nonspecific systemic exposure limit its application. To overcome these obstacles, our study explored the potential of non-carboxylated and carboxylated mesoporous carbon nanoparticles (MCN) as advanced drug carriers for bicalutamide (MCN/B and MCN-COOH/B). The physicochemical properties and release behaviour were thoroughly characterized. Functionalization with carboxylic groups significantly improved wettability, dispersion stability, as well as loading efficiency due to enhanced hydrogen bonding and π–π stacking interactions. Moreover, all systems exhibited sustained and near-infrared (NIR) triggered drug release with reduced burst-effect, compared to the release of free bicalutamide. Higher particle size and stronger drug–carrier interactions determined a zero-order kinetics and notably slower release rate of MCN-COOH/B compared to non-functionalized MCN. Cytotoxicity assays on LNCaP prostate cancer cells demonstrated that both MCN/B and MCN-COOH/B possessed comparable antiproliferative activity as free bicalutamide, where MCN-COOH/B exhibited superior efficacy, especially under NIR exposure. These findings suggest that MCN-COOH nanoparticles could be considered as a prospective platform for controlled, NIR-accelerated delivery of bicalutamide in prostate cancer treatment.

## 1. Introduction

Cancer is still the leading cause of mortality worldwide, affecting millions of people and causing serious problems to healthcare systems [1]. Current treatment strategies include a variety of methods, such as surgery, radiotherapy, chemotherapy, photodynamic therapy, as well as photothermal therapy (PTT) [2]. Chemotherapy, which utilises chemical substances to destroy tumour cells, has long been a key part of cancer treatment [3]. However, its lack of specificity often affects healthy cells as well, leading to serious side effects [3,4]. This determines the need for searching for targeted drug delivery systems.

Photothermal therapy is a physical treatment method that transfers light into heat to eliminate cancer cells [4,5]. Employing near-infrared (NIR) light (700–1100 nm) for this aim is distinctly helpful, since it can penetrate deep into tissues without having a serious risk of affecting healthy cells [3]. The efficacy of PTT can be augmented through the utilization of some nanomaterials, such as metal and carbon-based structures [2]. The combination of PTT with chemotherapy, known as chemo-photothermal therapy, acts as a powerful method, providing both heat and chemotherapeutic agents directly to tumour cells [2,5]. This synergistic approach makes cancer cells extremely sensitive to treatment, reduces drug resistance and chemotherapeutic dose, and minimizes damage to healthy tissues and systemic side effects [3,6,7].

Recent trends have been focused on developing nanosystems possessing both chemo- and photothermal effects [8]. Carbon-based NIR-responsive materials, including carbon nanotubes [3], graphene oxide, and mesoporous carbon nanoparticles (MCNs), offer considerable potential in chemotherapy [3]. However, their disadvantages, related to premature and rapid drug release, limit their therapeutic efficacy [2]. These challenges have triggered the need for developing a smart delivery system capable of selectively releasing drugs with improved effectiveness [2].

Among these, mesoporous carbon nanoparticles have gained significant attention due to their exceptional physical and chemical properties [5,7,9]. Compared to some organic-based nanoparticles, inorganic carbon nanomaterials are more stable, inert, and modifiable. MCNs, in particular, possess large specific surface areas, high pore volumes, tunable pore structures, and adjustable surface morphology, making them perfect as drug carriers [3,10,11]. They also demonstrate excellent biocompatibility, high loading capacities, especially for drugs with low hydrophilicity, as well as strong interaction capability with aromatic drugs, through π–π stacking [12,13,14]. Different researchers evaluated the strong photothermal conversion efficiency of mesoporous carbon material in their studies, both in vitro and in vivo cells [2,3,5,15,16,17,18]. Furthermore, the mesoporous carbon matrix offers excellent protection during delivery and enhances solubility [19] and membrane permeability for poorly soluble drugs, especially those belonging to class II of the Biopharmaceutic Classification System (BCS), characterized by low solubility and high permeability [20,21].

Such systems could achieve high drug-loading efficiency, cancer cell specificity, and NIR-triggered drug release via utilization of careful design [22,23]. However, the surface hydrophobicity of MCN, as well as poor wettability and dispersion stability, often leads to aggregation and sedimentation in aqueous environments, hampering their use in physiological conditions [5,12]. This obstacle also provokes premature drug release, limiting its bioavailability and effectiveness in vivo [7,16]. To meet these problems, modifying the surface morphology of MCNs is essential for ensuring better dispersibility in body fluids as well as improved uptake by target cells [7,13]. Functionalising the particle surface with different groups, such as –COOH, -NH_2_, PEG (polyethylene glycol), and PVP (polyvinylpyrrolidone), can successfully close the pore entrances, preventing premature drug release and improving stability during circulation [13]. Moreover, functionalization could also facilitate the control over the drug release by maintaining the therapeutic drug level for an extended period of time and reducing the fluctuations of drug concentration after oral administration [24].

Bicalutamide (B), an orally administered non-steroidal anti-androgen drug, is commonly used as first-line treatment of advanced prostate cancer due to its long half-life and tolerable side effects [25,26,27]. However, its low water solubility and suboptimal bioavailability after oral administration (drug class II of BCS) limit its ability to penetrate through biological membranes, thus reducing its therapeutic potential [26,28]. Another limitations of bicalutamide therapy are related to extensive first-pass metabolism and a high binding affinity to plasma proteins [25]. Considering the above-mentioned advantages of MCN as well as the aromatic and hydrophobic structure of bicalutamide, the aim of the present study is to explore the potential of carboxylated and non-carboxylated MCN as oral drug carriers of bicalutamide for combined chemo-photothermal therapy of prostate cancer. This study investigated the characteristics of MCN and the influence of functionalization, focusing on their drug-loading capabilities and controlled release profiles. In vitro experiments with prostate cancer cells (LNCaP) evaluated their cytotoxicity as well as how NIR light irradiation affects them.

## 2. Results and Discussion

### 2.1. Determination of Encapsulation Efficiency (EE%) and Loading Capacity (LC%)

Based on our previous experience, as well as a profound literature review, non-functionalized mesoporous carbon nanoparticles (MCNs) were successfully carboxylated (MCN-COOH) and subsequently loaded with the API, as detailed in the Methods section. The obtained non-carboxylated and carboxylated systems, both loaded with bicalutamide, were named MCN/B and MCN-COOH/B, respectively.

The encapsulation efficiency and the drug loading capacity are important parameters from both therapeutic and economic reasons. The encapsulation efficiency (EE%) is defined by the concentration of the API (bicalutamide in this case) found in a certain amount of nanoparticles over the initial amount of API used for loading. In the present study, EE% was determined indirectly by calculation of the difference between the initial amount of bicalutamide used for loading and the amount found in the supernatant after centrifugation and washing [29]. Loading capacity (LC%) represents the amount of API loaded per unit weight of the nanoparticles, indicating the percentage of mass of the nanoparticles that is due to the loaded drug. The results are presented in Table 1.

The presence of non-covalent interactions of π–π stacking between the carriers and the API was responsible for the high drug loading of both formulations—MCN/B and MCN-COOH/B [5]. However, both EE% and LC% were significantly higher (*p* < 0.05) for MCN-COOH/B (48.53% LC%) compared to MCN/B (41.08% LC%). This improvement after carboxylation was attributed to the dual contribution of π–π stacking and hydrogen bonding, which enhances bicalutamide interaction with the functionalized nanoparticle matrix. Hydrogen bonding between the functional groups in bicalutamide and the carbonyl (C=O) groups of the carboxylated nanoparticles offered new binding sites of bicalutamide on the nanoparticle surface, leading to almost complete encapsulation of bicalutamide in MCN-COOH (see Section 2.2). In comparison, the loading of the API into the bare nanoparticles could be associated only with simple π–π stacking between MCN and bicalutamide [5], leading to slightly poor drug loading. Comparable results about similar mesoporous materials were also reported by other researchers [5,30,31,32].

### 2.2. Fourier-Transform Attenuated Total Reflection Infrared (ATR-FTIR) Spectroscopy

The successful loading of bicalutamide, as well as its potential interactions with the carriers, were confirmed using ATR-FTIR spectroscopy. The obtained spectra are presented in Figure S1 (Supplementary Material). Table 2 includes detailed data on spectra peaks and values.

Infrared (IR) spectra of MCN and MCN-COOH were characterized by the presence of stretching vibrations of aromatic C=C bonds around 1587–1588 cm^−1^, resulting from the aromatization of MCN and the sp^2^-hybridized carbon framework [33]. The appearance of the strong absorption bands at 1687 cm^−1^ and 1256 cm^−1^ in the MCN-COOH spectrum, corresponding to the stretching vibrations of the carboxyl group’s C=O and C-O bonds, respectively, confirmed the successful carboxylation of the parent MCN nanoparticles [7].

The IR spectrum of bicalutamide was characterized by numerous distinct absorption peaks. The O-H stretching vibration appears at 3597 cm^−1^, and N-H stretching at 3336 cm^−1^, while bands at 2231 cm^−1^ and 1696 cm^−1^ corresponded to C≡N and C=O stretching, respectively [34,35]. The aromatic and aliphatic C-H asymmetric and symmetric stretching bands were observed at 3070 cm^−1^, 2975 cm^−1^, and 2935 cm^−1^, respectively [36]. The C-F stretching of the monofluorobenzene ring was prominent at 1231 cm^−1^, while CF_3_ showed a strong absorption at 1126 cm^−1^. The aromatic C=C stretching vibrations were noted at 1602 cm^−1^ and 1591 cm^−1^ [34]. The intense band at 1327 cm^−1^ was due to the presence of the S=O group [34,37]. In-plane bending vibrations βCH, βCH_3_, and βHCH appeared at 1514 cm^−1^, 1503 cm^−1^, and 1494 cm^−1^, respectively [36]. 

In the spectrum of MCN/B, a slight reduction in the intensity of bicalutamide characteristic peaks was assigned, without significant shifts, proving the absence of strong chemical interactions. Conversely, in the spectra of both non-carboxylated and carboxylated nanoparticles loaded with bicalutamide, a band broadening and shifting in the aromatic C=C stretching region (~1500–1600 cm^−1^) were evident. These spectral changes suggested successful entrapment of bicalutamide via non-covalent π–π stacking interactions [5]. π–π stacking refers to non-covalent interactions between π-orbitals of aromatic systems [38]. In this context, such interactions occurred between the phenyl rings of bicalutamide (acting as π-electron-rich systems) and graphitized or aromatic carbon domains (sp^2^-hybridized) of mesoporous carbon nanoparticles (both carboxylated and non-carboxylated). These interactions were key contributors to the efficient drug loading and retention of bicalutamide within the nanoparticle pores and on the surface [5,39].

In addition to π–π stacking, the IR spectrum of MCN-COOH/B displayed slight shifts in the peaks associated with the hydroxyl and amide groups. These shifts may be attributed to possible non-covalent hydrogen bonding between the free hydroxyl and amide groups of bicalutamide (acting as donors) and the carbonyl groups of the carboxylated nanoparticles (acting as acceptors). The coexistence of hydrogen bonding and π–π stacking was likely responsible for the observed improvements in encapsulation efficiency (EE%) and loading capacity (LC%) for the MCN-COOH/B formulation.

### 2.3. Dispersion Study of MCN and MCN-COOH

As already discussed in the introduction, one of the biggest limitations associated with carbon materials is their surface hydrophobicity as well as poor wettability, leading to aggregation and sedimentation in aqueous environments. The stability issues could hinder their use in physiological conditions [5,12]. There are many pieces of literature data demonstrating that modifying the surface properties and morphology of MCN ensures better dispersibility in body fluids [7,13,40]. For the above-mentioned reason, measurement of the dispersion stability is of great importance during research and development of new drug delivery systems. 

**Figure 1:**
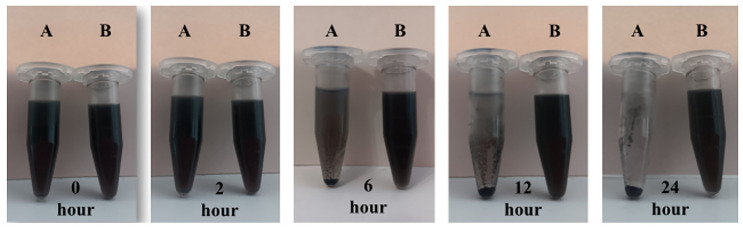
Dispersion stability photos of A. MCN and B. MCN-COOH in phosphate buffer with pH 6.8 at different times—0 h, 2 h, 6 h, 12 h, and 24 h.

The results of the performed dispersion study of non-modified MCN as well as carboxylated MCN-COOH nanoparticles in distilled water are presented in Figure 1. After 10 min of sonication, both compositions were dispersed uniformly and preserved their dispersion stability for the first hour of the experiment, since no evidence of sedimentation was observed. Two hours later, MCN (sample A) showed the initial form of aggregation and sedimentation from the medium, while MCN-COOH (sample B) was still homogeneously dispersed. The processes of aggregation and sedimentation for unmodified nanoparticles continued during time, and at the 24th hour, almost complete sedimentation was noted, indicating that unmodified MCN were poorly water-soluble and possessed extreme hydrophobicity. At the same time, carboxylated nanoparticles (MCN-COOH–sample B) demonstrated excellent dispersibility, since they retained their uniform dispersion characteristic until the end of the experiment. The results suggested that carboxylation successfully modified the surface of MCN nanoparticles, increasing their hydrophilicity and wettability, preventing aggregation and sedimentation, which improved their dispersion stability in aqueous environments. Similar observations have been published previously [3]. The results from the dispersion study were in accordance with the results obtained from the DLS experiment (about PDI and zeta potential), given in the next section.

To strengthen the observation about nanoparticles’ stability [41,42,43,44], additional DLS measurements at different time intervals (e.g., 0, 2, 6, 12, and 24 h) as well as turbidimetric studies were performed (data are presented in Supplementary Material, Figure S2). An increase in size and/or PDI and a decrease in the absolute value of Z-potential over time is evidence for aggregation. Stable nanoparticles should maintain a consistent size, low PDI, and Z-potential above 30 mV (as absolute value) over time [45]. The suspension is considered to be stable when the measured absorbance spectra is less than 10% of the initial reference peak up to 72 h [46,47].

Non-functionalized MCNs demonstrated progressive growth in size and PDI over time, which is an indicator of aggregation and sedimentation. The aggregation over time is likely due to their critical zeta potential value (between −9 and −7 mV), which provides insufficient electrostatic repulsion to neutralize attractive van der Waals forces, in accordance with DLVO theory [48]. The inherently hydrophobic surface of MCNs encourages particle–particle attraction in aqueous environmens, further causing clustering via nonpolar interactions. Without functional coatings, MCNs lack steric protection to prevent aggregation, leading to poor colloidal stability. Another piece of evidence for MCN’s gradual sedimentation is the reduced turbidity till 10% at 72 h, which is consistent with the visual sedimentation noted in the manuscript.

The size of carboxylated MCN (MCN-COOH) remained nearly constant over 24 h (typical for dispersions in aqueous media and could be even considered within error range) and combined with the stable and low PDI value (<0.2) suggested consistent monodispersity. MCN-COOH uses surface–COOH groups (hydrated and negatively charged), reflected in a strongly negative Z-potential (above −35 mV). This provides good electrostatic stabilization and repulsion between particles as well as steric hindrance, keeping them well-dispersed, slowing sedimentation, and enhancing colloidal stability. Turbidity remained above 90% at 72 h, further supporting that MCN-COOH improved dispersion behavior.

The dispersion stability studies were conducted after the initial sonication to ensure reproducible dispersion in vitro and could not serve as a model to mimic in vivo dispersion conditions. Future experiments would evaluate the stability of MCN and MCN-COOH systems in simulated oral administration conditions. However, the results from the conducted stability studies suggest greater potential of MCN-COOH for spontaneous dispersion in the gastrointestinal tract without the need for external force.

### 2.4. Dynamic Light Scattering (DLS) Analysis

Table 3 summarizes the information about the data from DLS analysis in different media (deionized water and buffers with pH 1.2, pH 5.0, and pH 6.8). The results about size and polydispersity index (PDI), as well as zeta potential (Z-potential), are in good accordance with the literature about both non-carboxylated [11,40,49] and carboxylated MCN [5,7,35].

Negative Z-potential values of both types of non-loaded mesoporous carbon nanoparticles are typical for such types of mesoporous material. After carboxylation of MCN, a significant increase in Z-potential value was observed (from −9.02 mV for MCN to −38.05 mV for MCN-COOH), as a result of the negative deprotonated carboxyl groups on the surface of carboxylated nanocarriers, which were ionized in aqueous solutions [11]. This observation could also serve as proof for the efficient carboxylic functionalization of the carbon carriers [16]. The strongly negative Z-potential value of carboxylated (−38.05 mV), compared to unmodified (−9.02 mV) nanoparticles in deionized water, was a prerequisite for the observed improved dispersion stability of MCN-COOH, since the value of ±30 mV is considered as evidence of sufficient colloidal stability [45,48,50].

Drug loading resulted in a slight decrease in the absolute value of the zeta potential for both non-carboxylated and carboxylated nanoparticles. This reduction was likely due to partial deposition of bicalutamide on the surface, which masks or neutralizes some of the surface charges. As a poorly water-soluble molecule with a high pKa (~12), bicalutamide remains largely neutral in distilled water and does not contribute significantly to the net surface charge of the system [27]. The slightly decreased Z-potential absolute value after the drug loading procedure could be considered as evidence of successful encapsulation of bicalutamide. Regardless of this, MCN-COOH/B possessed sufficient colloidal stability, since its Z-potential value is high enough (−34.13 mV).

**Figure 2:**
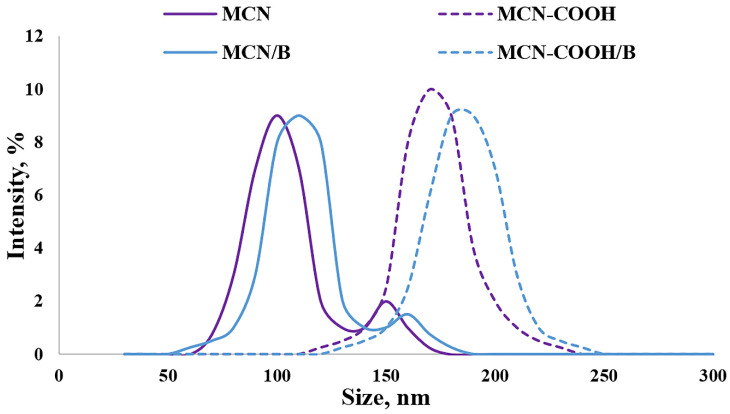
Size-distribution diagram for determination of Z-average diameter (hydrodynamic diameter) of the mesoporous carbon nanoparticles measured with DLS, presented by % intensity.

The Z-average diameters (hydrodynamic diameters) of all of the prepared formulations in deionized water are presented in Figure 2. The data about the size of all of the prepared samples demonstrated sufficiently small particle sizes (below 200 nm) suitable for oral administration [51]. Even though carboxylation led to particle size increase, no agglomeration was observed, as evidenced by the decreasing value of the PDI (lower than 0.3), indicating a narrow distribution. An increase in particle size in this case could be a consequence of successful carboxylation on the surface of nanoparticles [3]. A decrease in the PDI value indicated that carboxylation increased the dispersion stability of MCN-COOH and MCN-COOH/B. The higher PDI (above 0.3) as well as the presence of a weak intensity second peak in the size distribution diagram of MCN and MCN/B suggested moderate polydispersity and could serve as evidence for aggregation, due to the hydrophobic nature of MCN and MCN/B and bad wetting and dispersing properties (see dispersion study). The loading procedure was considered to be appropriate, since there was just a weak tendency for an increase in size, and the PDI was kept below 0.3, indicating good distribution.

Media pH has a different influence on non-functionalized and carboxylated MCN nanosystems. The results demonstrated typical pH-dependent behavior for carboxylated nanoparticle systems, since both MCN-COOH and MCN-COOH/B exhibited reduced particle sizes and lower polydispersity indices (PDI) at pH 6.8, with favourable zeta potential values, indicating high colloidal stability under intestinal conditions. This could be attributed to the deprotonation of surface –COOH groups, which increases electrostatic repulsion and prevents aggregation. In contrast, under acidic conditions (pH 1.2), a significant increase in particle size and PDI could be observed, caused by protonation of carboxyl groups and reduction in surface charge (zeta potential decrease), resulting in partial aggregation. Since non-functionalized MCN and MCN/B samples do not possess ionizable surface groups, they exhibited relatively unchanged size, PDI, and zeta potential values across all pH levels. These findings could serve as proof of the importance of carboxyl functionalization for improving nanoparticle stability and achieving pH-responsive behavior relevant to oral drug delivery. Similar observations about different pH-responsive behaviour of carbon nanosystems were also introduced by other researchers [52,53,54].

### 2.5. X-Ray Diffraction Analysis (XRD)

**Figure 3:**
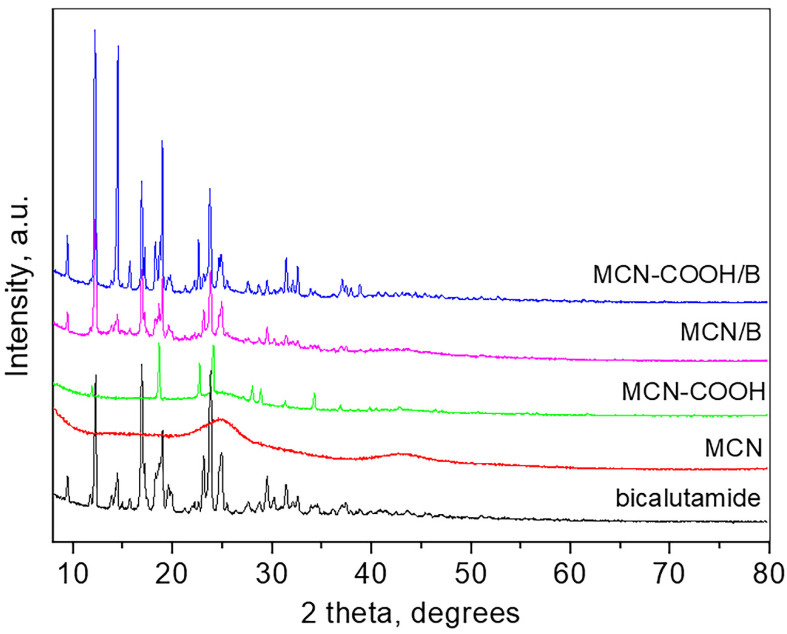
XRD patterns of bicalutamide, MCN, MCN-COOH, MCN/B, and MCN-COOH/B.

Wide-angle XRD patterns of bicalutamide, MCN, carboxylated MCN, and the drug-loaded MCN and MCN-COOH are presented in Figure 3.

The pattern of bicalutamide shows the high crystalline nature of the compound with sharp and strong peaks which positions are consistent with the bicalutamide monoclinic form I (SG P21/c) and the unit cell parameters determined are a = 14.893 (4) Å, b = 12.220 (2) Å, c = 10.480 (2) Å, β = 104.77 (8), respectively, close to those reported [55]. The crystallite size is 68 nm. The pattern of MCN is typical for amorphous carbon material, showing two broad humps at around 25 and 43° 2θ. The pattern of carboxylated MCN-COOH shows several strong peaks superimposed on the amorphous pattern, which are attributed to the presence of crystalline NH_4_HSO_4_ (PDF card 00-025-0034) due to the preparation procedure. The pattern of the bicalutamide-loaded MCN, as well as that of the bicalutamide-loaded MCN-COOH, are similar and consist only of peaks related to bicalutamide (unit cell parameters for MCN/B: a = 14.843 (4) Å, b = 12.227 (2) Å, c = 10.497 (2) Å, β = 104.72 (1); unit cell parameters for MCN-COOH/B: a = 14.949 (2) Å, b = 12.219 (1) Å, c = 10.483 (3) Å, β = 104.71 (2)) and amorphous carbon. The crystallite size of MCN/B is 77 nm, while that of MCN-COOH/B is 93 nm. The low-angle part measurements of all samples did not show any long-range pore ordering.

### 2.6. Nitrogen Adsorption

**Figure 4:**
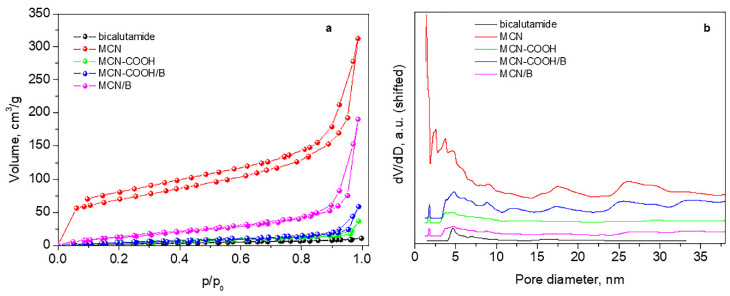
Nitrogen adsorption–desorption isotherms (**a**) and pore-size distributions (**b**).

Figure 4 and Table 4 present adsorption–desorption isotherms, pore-size distributions, and texture parameters of the studied materials.

All samples present mixed II type and IV type isotherms according to IUPAC classification (pseudo-II type) characteristic of non-porous or macroporous material with hysteresis loop H3 (Figure 4a). Hysteresis H3 reveals wide-range mesopores, which are confirmed by their pore-size distribution curves (Figure 4b).

The initial sample MCN possesses a relatively high specific surface area and large total pore volume with a small amount of micropores (Table 4). After deposition of bicalutamide, there is a large decrease in the specific surface area and almost a twofold reduction in the volume, accompanied by the disappearance of micropores and an increase in the average pore size, due to blocking or filling of pores of smaller sizes by the drug.

The carboxylation of the material leads to a drastic decrease in both the specific surface area and the total pore volume, accompanied by an increase in the average pore size. This is due to a residual amount of ammonium bisulfate, recorded in the XRD of MCN-COOH, which deteriorates the porous texture of the material. For this reason, an accurate assessment of the texture of this sample cannot be made. Ammonium bisulfate is formed during the carboxylation process and is not eliminated by washing with ethanol due to its poor solubility in it.

However, in the process of bicalutamide loading, this ammonium bisulfate dissolves in the added water, and, after centrifugation, the filled or blocked pores are liberated, and, thus, despite the deposition of the drug, the specific surface area and the total pore volume increase.

### 2.7. Transmission Electron Microscopy (TEM)

**Figure 5:**
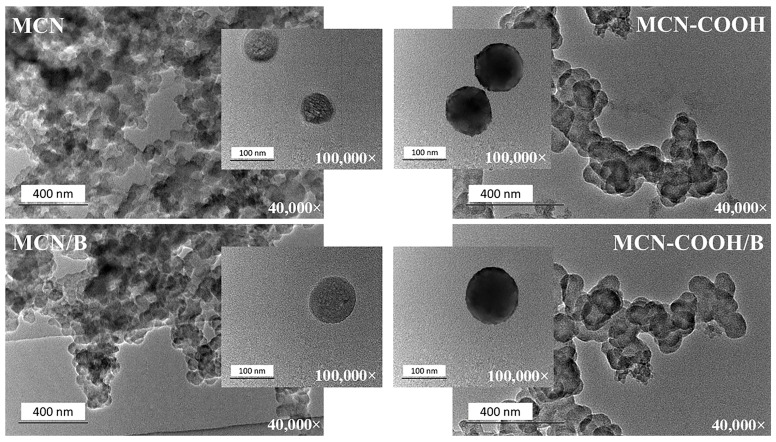
TEM micrographs of MCN, MCN/B, MCN-COOH, and MCN-COOH/B taken at 40,000× and 100,000× magnifications.

Transmission electron microscopy (TEM) was applied to prove the mesoporous structure and morphology of the prepared nanoparticles and to investigate the possible changes upon carboxylation as well as drug loading. TEM images of all of the prepared formulations taken under 40,000× and 100,000× magnifications are presented in Figure 5. The slight inconsistency between nanoparticle morphologies in the lower and higher magnification TEM images can be attributed to sample variability before TEM observation. During sample preparation, drying caused nanoparticles to aggregate or cluster on the grid, resulting in overlapping, which was visible at low magnifications, whereas high-magnification images appeared to be more uniform, as they were selected from areas with well-dispersed individual particles [56,57,58,59]. Regardless of the magnification used, all of the compositions exhibited a uniform spherical morphology with a narrow worm-like mesopore channel, confirming the ordered structure of mesoporous carbon nanoparticles [7,16]. Carboxylation did not change the overall morphology of the nanoparticles and only slightly increased the contrast, possibly due to the introduction of oxygen-rich functional groups [60,61]. No significant changes were observed after drug-loading procedures, proving that mesoporous carbon materials possess high mechanical stability.

The DLS registered sizes (Table 3) of all of the compositions were comparatively larger than those obtained by TEM analysis (in the range of 70 nm–120 nm). This phenomenon was because of the specificity of the samples’ preparation between the two methods. The TEM samples were measured in a dry state, whereas DLS measured hydration diameters of the samples, which included the surface hydration layer around particles dispersed in distilled water [5].

### 2.8. In Vitro Drug Release Study

**Figure 6:**
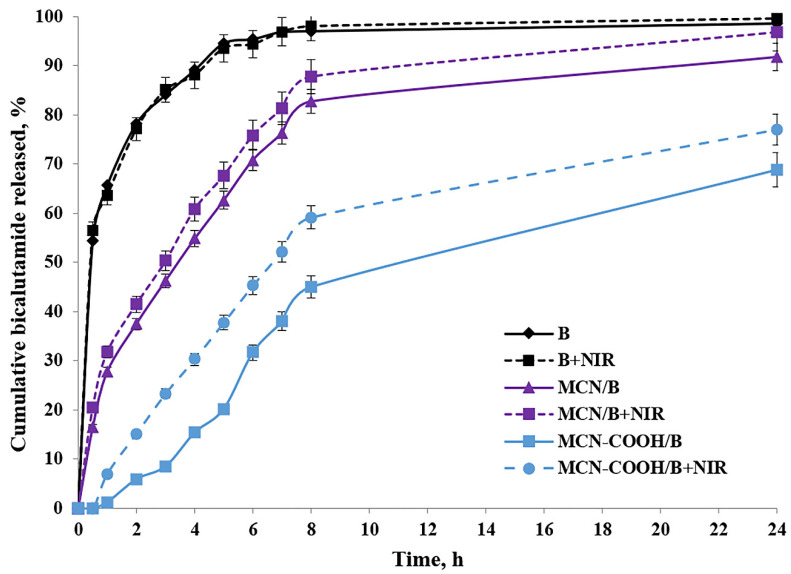
In vitro release profiles of free bicalutamide (B) and loaded nanoparticle formulations MCN/B and MCN-COOH/B with or without NIR light; mean ± SD; n = 3.

After oral administration, MCN-COOH/B nanoparticles are anticipated to navigate the gastrointestinal tract, where their improved aqueous dispersibility and stability promote more efficient interaction with the intestinal epithelium. Although most of the drug is expected to be absorbed as a free molecule after release from the carriers, recent studies demonstrated that nanosystem formulations can enable the oral bioavailability of bicalutamide by improving solubility and interaction with intestinal membranes [26,62]. Besides this, the intact nanoparticle uptake cannot be excluded as well. Ran et al. [13] demonstrated size-dependent oral absorption of polymer-functionalized mesoporous carbon nanoparticles through endocytosis or paracellular transport pathways. Although the nanoscale size and carboxylated surface have the potential to support some interaction with the intestinal epithelium, we acknowledge that the current study does not directly prove the transport across the gastrointestinal barrier and the systemic delivery of intact MCN-COOH/B remains hypothetical at this stage. Upon absorption, bicalutamide is expected to be released in a controlled manner, enhancing systemic bioavailability while reducing burst effects compared to free drug. Nanoparticle fate post-absorption typically involves systemic distribution, gradual biodegradation, and eventual elimination; however, specific biodistribution and degradation pathways depend on nanoparticle physicochemical properties and require further in vivo investigation using gastrointestinal epithelial models such as Caco-2 cells and pharmacokinetic studies. As an orally administered drug, the usual media for bicalutamide release determination were chosen to be a standard acidic buffer with pH 1.2, as well as Sorensen phosphate buffer with pH 6.8. Additionally, the release behavior of bicalutamide was studied in a citrate buffer with pH 5.0 to simulate the microenvironment conditions in tumor cells [63]. All in vitro dissolution studies were conducted with or without 3 min of near-infrared (NIR) light irradiation. The dissolution of free bicalutamide substance served as a comparison. The results about the cumulative bicalutamide release from both types of mesoporous carbon nanoparticles (non-carboxylated MCN/B and carboxylated MCN-COOH/B) in pH 5.0 medium, as well as the data about the T_50_ value in all media types, are presented in Table 5 and Figure 6.

The release of bicalutamide from mesoporous carbon nanoparticles (MCNs) was supposed to depend on: the pore structure (pore size and surface area) of the carriers, pH-dependent solubility of bicalutamide, as well as nanoparticles’ surface properties, which could lead to drug–nanoparticle interactions. 

Bicalutamide is, overall, a neutral, weakly acidic API, which could participate in hydrogen bonding. Given its high pKa (~12), bicalutamide remains predominantly neutral under all tested pH conditions (1.2–6.8), making strong electrostatic interactions with the nanoparticle surface unlikely. Thus, among pH 1.2, 5.0, and 6.8, no significant difference was expected, and bicalutamide would remain poorly soluble in all cases. Additionally, faster release was expected in a pH environment (above pH 1.2), where bicalutamide had higher solubility and weaker drug–nanoparticle interactions, and vice versa [28].

Mesoporous materials provide a confined environment where drug molecules are physically entrapped within the pores due to hydrophobic interactions and π–π stacking. The small pore size and tortuous diffusion path slowed down the release of bicalutamide from both nanoparticle formulations, resulting in sustained release, compared to the diffusion of the free bicalutamide substance in all media. Similar data could be found from different research studies [10,64]. Considering the neutral charge of bicalutamide in physiological conditions, we suggested that it should remain long enough inside the mesochannels of MCN in an aqueous environment, preventing significant leakage, and be released predominantly in the hydrophobic regions of the tumor cell compartments after the uptake [65,66,67].

Carboxylated MCNs have surface -COOH functional groups, which allow the formation of hydrogen bonds and, eventually, electrostatic interactions (depending on pH conditions). Therefore, drug release from carboxylated nanoparticles should be governed by both diffusion and drug-nanoparticle interactions [5]. These hydrogen bonds could contribute to the adsorption or binding of bicalutamide inside the pores, slowing down the drug’s diffusion, leading to even more sustained release and reduced burst-effect compared to non-functionalized MCNs, as previously shown [68]. Moreover, carboxylated MCNs were more hydrophilic, which may increase bicalutamide retention in the pores due to interactions with water molecules and the formation of a hydration layer. Vice versa, non-functionalized MCNs had a more hydrophobic pore surface, making them less likely to retain bicalutamide strongly (it was loosely held), leading to faster release. According to the theory of diffusion, the slightly larger particle size of MCN-COOH/B may have contributed to the slower release rate due to increased diffusion path length, compared to the non-carboxylated. All this reflected a higher value of T_50_ (slower release) for MCN-COOH/B compared to MCN/B in all media types.

Since non-functionalized MCNs have a neutral, hydrophobic carbon surface without ionizable functional groups, and at the same time, bicalutamide itself is also neutral in the studied pH range (1.2–6.8), no strong pH-dependent electrostatic interactions or hydrogen bonds between the drug and the nanoparticle surface were expected. Thus, the pH-dependent release behavior of bicalutamide loaded in MCN/B was unlikely to be observed. As a result, release was expected to be primarily diffusion-driven rather than pH-sensitive. These reflected in a practically insignificant difference in the dissolution of free API as well as the release of bicalutamide from MCN/B into different pH media. 

The factors influencing bicalutamide release in different pH media from carboxylated MCN were more complicated, since the dissolution processes were additionally affected by the presence of carboxyl groups on the nanoparticles’ surface. At pH 1.2 (acidic conditions like in the stomach), carboxyl groups remain protonated (-COOH^−^), reducing electrostatic repulsion and causing nanoparticles aggregation, which slowed down drug release. Because bicalutamide is neutral at pH 1.2, it can only form weak hydrogen bonds, and the lack of strong ionic interactions, combined with lower solubility and nanoparticle aggregation, likely hinders its release under these acidic conditions. At pH 6.8 (intestinal conditions) and pH 5.0 (tumor microenvironment), carboxyl groups were mostly deprotonated (-COO^−^), keeping nanoparticles well dispersed, potentially allowing better drug diffusion. Since bicalutamide remained neutral, there was no strong binding interaction between the drug and the nanoparticle. The negative charges on the nanoparticle surface could repel each other, enhancing drug diffusion. As a result, the values of T_50_ for MCN-COOH/B at these pH media were lower compared to those in pH 1.2, which corresponded with faster release in these media.

To study the effect of NIR light irradiation on the release of bicalutamide, the dissolution studies were performed in the absence or presence of NIR light irradiation for 3 min (under 808 nm with the power density of 3 W) after taking samples at each time interval. Drug release from both types of carbon-based nanosystems was significantly accelerated upon brief NIR irradiation. This result might be attributed to the photothermal capacity of carbon materials to absorb the light energy and convert it into heat energy after irradiation, which increased the local temperature [7,40]. Molecular thermal motion, expansion of the pores, and weakening of drug–carrier interactions (such as π–π stacking and hydrophobic forces) likely facilitated drug release from both MCN/B and MCN-COOH/B systems [5]. Such an effect was not observed in the dissolution of free API, since the difference between the values of T_50_ of non-irradiated and irradiated samples was practically the same in all media types. Thus, these results indicated that the NIR-induced heat could trigger the drug release and, thus, led to an increased chemotherapeutic efficacy by minimizing the side effects [40].

### 2.9. Release Kinetics Study

Four types of mathematical models, including zero-order, first-order, Higuchi, and Korsmeyer–Peppas (K–P), were used to study the release kinetics of non-carboxylated and carboxylated mesoporous carbon nanoparticles loaded with bicalutamide in buffer media with pH 5.0, with or without NIR-irradiation. The results of the fitting, presented in Table 6, suggested that the drug release mechanism was significantly influenced by the surface functionalization of mesoporous carbon nanoparticles (MCNs). The release kinetics from the non-carboxylated nanoparticles (MCN/B and MCN/B + NIR) followed the Higuchi model, since the correlation coefficients (R^2^) were greater than those of the other models. This suggested that bicalutamide release from MCN/B was controlled primarily by diffusion from the porous matrix. The drug molecules were likely entrapped within the mesoporous structure and gradually diffused out over time [69,70]. Drug release was faster initially and then slowed down as the concentration gradient decreased. The diffusion-driven release of the API from non-carboxylated nanoparticles could also be confirmed by the K-P model [71]. The value of the release exponent “n” for these formulations was around 0.5, which corresponded to Fickian diffusion in slab-like matrix systems [71]. Unlike non-carboxylated, carboxylated mesoporous carbon nanoparticles with bicalutamide (MCN-COOH/B and MCN-COOH/B + NIR) exhibited zero-order release kinetics. This model suggested that the drug was released at a constant rate, independent of concentration [72,73] and governed by both diffusion and drug–nanoparticle interactions. The different release kinetics mechanism of the carboxylated formulation was probably caused by the presence of carboxyl functional groups, which were able to enhance drug binding through hydrogen bonding or electrostatic interactions in pH 5.0 buffer media. Thus, carboxylation could regulate release, preventing a rapid burst release and ensuring steady, sustained drug delivery. Interpretation of the release exponent (n) from the K-P model in the case of zero-order kinetics is generally not appropriate, since zero-order release suggests a non-diffusion-controlled mechanism, independent of concentration [71].

NIR irradiation was not likely to have an impact on the release kinetics, as no significant changes in the R^2^ value or k were observed for the different models, compared to non-irradiated systems.

### 2.10. In Vitro Cell Viability Assays

**Figure 7:**
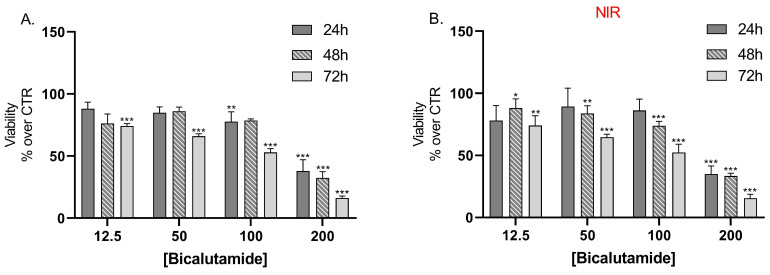
Effect of bicalutamide on LNCap cell line viability (**A**) and on NIR-irradiated LNCap cell line viability (**B**) after 24 h, 48 h, and 72 h treatment. Data are presented as mean values ± SEM. * *p* < 0.05; ** *p* < 0.05; *** *p* < 0.001 vs. CTR, one-way Anova.

Results from the cell viability assay showed that bicalutamide alone caused dose and time-dependent cytotoxic effects on LNCaP cells with no statistically significant differences in the effects between the groups, irradiated with NIR light, compared to the non-irradiated group of cells (Figure 7A,B).

**Figure 8:**
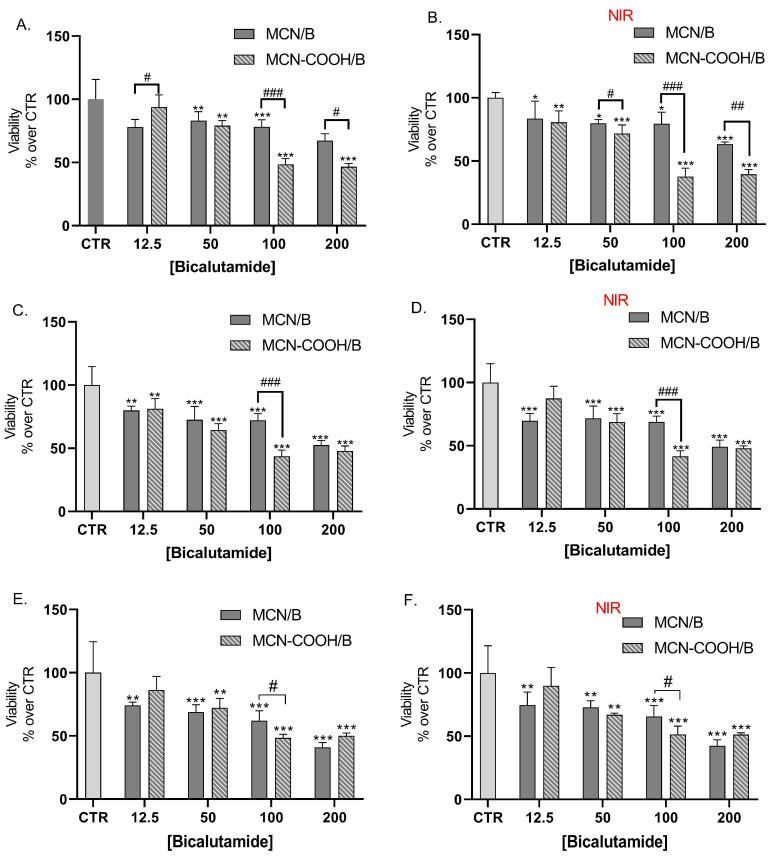
Effect of bicalutamide-loaded MCN/B and MCN-COOH/B in non-irradiated (**A**,**C**,**E**) and NIR-irradiated (**B**,**D**,**F**) LNCap cell line viability after 24 h (**A**,**B**), 48 h (**C**,**D**), and 72 h (**E**,**F**) treatment. Data are presented as mean values ± SD. * *p* < 0.05, ** *p* < 0.01, *** *p* < 0.001 vs. CTR, one-way Anova; # *p* < 0.05, ## *p* < 0.01, ### *p* < 0.001 multiple *t*-tests with Holm–Sidak correction.

LNCaP cells were also treated with bicalutamide, loaded in either MCN or MCN-COOH, in order to evaluate both the effects of drug loading and of particle functionalization (Figure 8A,C,E). Furthermore, we compared the effects of NIR irradiation on treated cells’ viability (Figure 8B,D,F).

Our results showed that loading of bicalutamide in both MCN and MCN-COOH preserved its cytotoxicity effects in LNCaP androgen-sensitive human prostate adenocarcinoma cells.

A major observation was that after 24 h treatment, at concentrations 100 µM and 200 µM, MCN-COOH/B caused significantly more pronounced effects than MCN/B. After 48 h and 72 h treatment, such differences were again observed, despite the difference in the cytotoxic effect being less pronounced and statistically significant at 100 µM. At most applied concentrations, both MCN/B and MCN-COOH/B treatments exerted similar effects, irrespective of whether they were irradiated with NIR or not. After 24 h of treatment with MCN-COOH/B, NIR irradiation resulted in increased cytotoxic effects at most concentrations. The effects of MCN-COOH/B treatment were stronger than those of MCN/B after shorter timeframes (24 h) with NIR irradiated cells. A probable explanation points to differences in the release kinetics of both systems in the context of NIR-irradiation. Initially, NIR provoked the release of bicalutamide to a higher degree in MCN-COOH/B. Later, at 48 h and 72 h, the remaining loaded fraction of the drug was likely decreased in MCN-COOH/B, but it was still steadily released by MCN/B.

Previously, it has been shown that loading in particulate drug-delivery systems is an effective strategy to improve bicalutamide pharmacokinetics-related properties while retaining or improving its pharmacological effectiveness in vitro. Loading in poly (D,L-lactide-co-glycolide) [74], peptide carriers [75], and silica particles [76] improved its antiproliferative effects towards LNCaP cells.

The overall increase in the cytotoxic effect of the loaded carbon carriers under NIR irradiation might be explained by the elevated temperature, which could increase the molecular thermodynamic movement and likely enhance cell membrane penetrability and the sensitivity to bicalutamide. These results demonstrate the potential application for cancer treatment [7]. However, further studies including in vivo pharmacokinetics and tumor growth inhibition assays will be needed to confirm that this observation is valid in the context of the complex tumor microenvironment and to adequately quantify the extent of dose-sparing effects.

**Figure 9:**
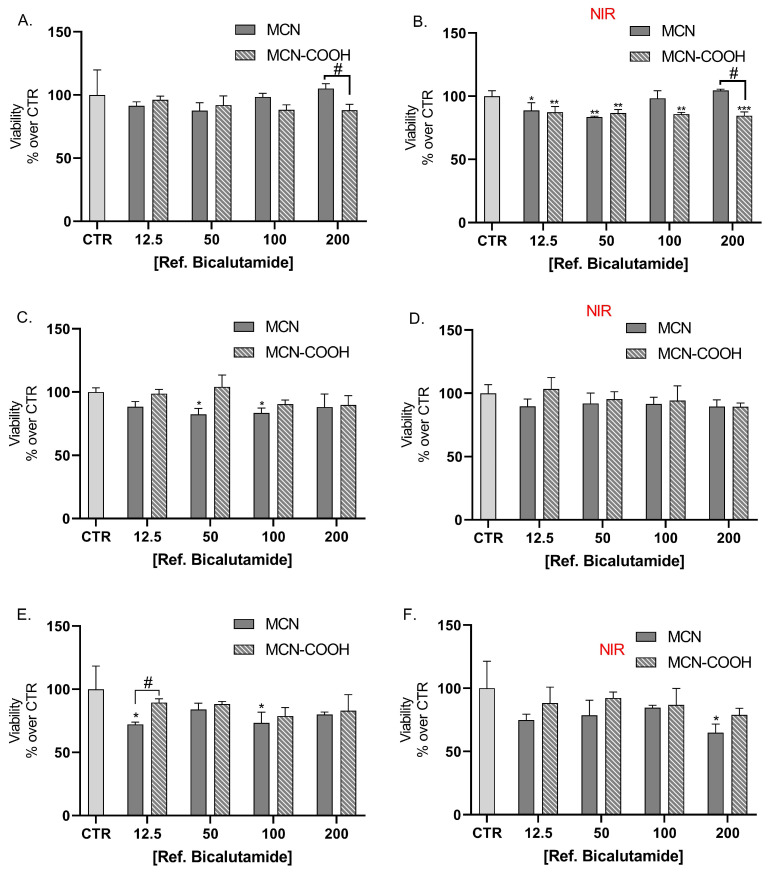
Effect of non-loaded MCN and MCN-COOH on LNCap cell line viability (**A**,**C**,**E**) and on NIR-irradiated LNCap cell line viability (**B**,**D**,**F**) after 24 h (**A**,**B**), 48 h (**C**,**D**), and 72 h (**E**,**F**) treatment, applied in the same concentration range as reported with the corresponding concentrations of bicalutamide in loaded MCN/B and MCN-COOH/B. Data are presented as mean values ± SEM. * *p* < 0.05, ** *p* < 0.01, *** *p* < 0.001 vs. CTR, one-way Anova; # *p* < 0.05 multiple *t*-tests with Holm–Sidak correction.

In order to exclude the possibility that the observed cytotoxic effects are not caused by bicalutamide, but the drug-delivery system, we further studied the effect of non-loaded MCN or MCN-COOH in the same concentration ranges as applied with non-loaded nanoparticles. Treatment of LNCaP cells with non-loaded drug delivery system did not cause dose-dependent toxicity, although some variation among the values in different groups was observed. This was valid for both the NIR-irradiated and non-irradiated groups (Figure 9). Overall, the MTT assay shows that both MCN and MCN-COOH alone did not exert considerable cytotoxic effects. The most pronounced difference due to NIR irradiation (15%) was observed with 200 µM MCN treatment, where either the viability scores were increased after 24 h (Figure 9B), likely due to the higher absorbance of the darker samples or decreased after 72 h (Figure 9F), because of the presence of weak cytotoxic effects. Overall, the tested non-loaded drug delivery systems, MCN and MCN-COOH, did not exert significant cytotoxic effects. This is in line with previous studies, which have found that surface-modified mesoporous carbon nanoparticles possess favorable biocompatibility, low hemolytic activity [2], and minimal acute toxicity in cellular and animal models [12]. However, further biocompatibility studies, including hemocompatibility and long-term in vivo safety assessments, will be required to fully confirm the suitability of the current drug-delivery systems for systemic therapeutic use.

## 3. Materials and Methods

### 3.1. Materials

Bicalutamide, sulfuric acid, and sodium lauryl sulphate (SLS) were obtained from MedChemExpress (Princeton, NJ, USA). Empty MCN nanoparticles, ammonium persulphate, acetone, HEPES (anhydrous ≥ 99.5%), sodium pyruvate, L-glutamine, fetal bovine serum (FBS), RPMI-1640, tripsin/ethylenediaminetetraacetic acid (EDTA), dimethylsulfoxide (DMSO), penicillin, streptomycin, and resazurin sodium salt were delivered from Sigma Aldrich (Merck KGaA, Darmstadt, Germany).

### 3.2. Carboxylation of Mesoporous Carbon Nanoparticles

Functionalization of empty MCN with carboxylic groups was performed via the wet oxidation method in a one-stage procedure [11]. The preheated 0.5 g MCN (at 120 °C for one hour) nanoparticles were incubated in a 30 mL water solution of ammonium persulfate (11.43% *w*/*w*) with 1.3 mL sulfuric acid under permanent stirring (180 rpm) and reflux for two hours at 70 °C [7,11]. The procedure continued with the separation of the carboxylated black MCN by centrifugation at 3000 rpm using a centrifuge (MPW-260 Instruments), washing with ethanol, and drying in a vacuum desiccator [2,3,5]. The obtained sample was named MCN-COOH.

### 3.3. Bicalutamide Loading into Non-Carboxylated and Carboxylated MCN 

The solvent-impregnation technique was used for bicalutamide loading [76]. The procedure started with incubation of 0.2 g (preheated at 120 °C for 1 h) MCN and MCN-COOH nanoparticles into 60 mL acetone solution of bicalutamide (3.5 mg/mL) under reflux at 37 °C and permanent stirring (180 rpm) for 24 h. Subsequently, 25 mL of water was added as a non-solvent, and acetone was evaporated by vacuum distillation. MCN/B and MCN-COOH/B nanoparticles were separated from water by centrifugation and dried in a vacuum desiccator. Bicalutamide supernatant was collected to measure drug-loading capacity (LC%) and encapsulation efficiency (EE%) by UV–vis spectrophotometer (Thermo Scientific Evolution 300, Thermo Fisher Scientific, Madison, WI, USA) at 270 nm.

### 3.4. Determination of Encapsulation Efficiency (EE%) and Loading Capacity (LC%)

The encapsulation efficiency (EE%) for bicalutamide from non-carboxylated and carboxylated MCN nanoparticles was carried out by calculation of the difference between the initial amount of the corresponding API (active pharmaceutical ingredient) used for loading (B_total_) and the amount found in the supernatant after centrifugation and washing (B_free_), based on Equation (1) [29]. In addition, the loading capacity (LC%) was calculated based on Equation (2) [77,78]. The assay of the API was determined by UV-vis spectrophotometry at 270 nm. The concentration of bicalutamide was calculated according to the standard curve (r^2^ > 0.991)

EE (%) = (B_total_ − B_free_) × 100/B_total_ (1)

LC% = (B_total_ − B_free_) × 100/weight of loaded MCN (2)

### 3.5. Fourier-Transform Attenuated Total Reflection Infrared (ATR-FTIR) Spectroscopy

Fourier-transform attenuated total reflection infrared (ATR-FTIR) spectra were evaluated by an FT-IR spectrometer, Nicolette 400 (Thermo Fisher Scientific, Waltham, MA, USA), using the KBr pellet method. Dried nanoparticles (non-loaded MCN and MCN-COOH and loaded with bicalutamide MCN/B and MCN-COOH/B) as well as the free API were mixed with potassium bromide to form a fine powder and observed over the spectral region 400–4000 cm^−1^ at a resolution of 4 cm^−1^.

### 3.6. Dispersion Study of MCN and MCN-COOH

A total of 10 mg of non-loaded MCN and MCN-COOH were measured and dispersed in 10 mL Sorensen phosphate buffer with pH 6.8 (1 mg/mL) and sonicated (Bandelin Sonoplus HD3100, Bandelin Electronics, Berlin, Germany) for 10 min at 75% amplitude. The samples were stored at room temperature, and the dispersion state was examined visually and photographed at predetermined time intervals: 0 h, 2nd hour, 6th hour, 12th hour, and 24th hour [40].

In order to straighten the stability observation of MCN and MCN-COOH (0.01 mg/mL), DLS measurements were taken after sonication for 10 min at 75% amplitude at the same time points, as the cuvettes were left undisturbed after the first time point.

Besides this, turbidimetric analysis was also performed according to the adapted protocol [79]. The MCN and MCN-COOH samples were dispersed in pH 6.8 phosphate buffer (0.01 mg/mL) and sonicated for 10 min at 75% amplitude to ensure homogeneity. The %turbidity at 600 nm was measured immediately (time 0) and after 2, 6, 12, 24, 48, and 72 h, as the samples were left undisturbed. 

### 3.7. Dynamic Light Scattering (DLS) Analysis

The average particle size, polydispersity index (PDI), and zeta potential of the empty (carboxylated and non-carboxylated) and loaded with the API (MCN/B and MCN-COOH/B) nanoparticles were determined using a Zetasizer Nano ZS (Malvern Instruments, Malvern, Worcestershire, UK). The samples were dispersed either in deionized water or media with pH 1.2 (hydrochloric acid buffer), pH 5.0 (citrate buffer), and pH 6.8 (sorensen phosphate buffer) at a 0.01 mg/mL concentration and measured at a scattering angle of 90° and 25°.

### 3.8. X-Ray Diffraction Analysis (XRD)

X-ray diffraction (XRD) patterns were obtained within the range from 5° to 80° 2θ (2θ step = 0.02°), counting time = 17.5 s/step on a Bruker D8 Advance diffractometer with a Cu Kα source (1.5406 Å, 40 kV, 40 mA) and LynxEye detector. The unit cell parameters and mean crystallite sizes were determined with Topas 4.2. The low-angle part was collected in the 0.4–6° 2θ range (step 0.02° 2θ) with the help of an additional special knife-edge.

### 3.9. Nitrogen Adsorption

The texture of the parent MCN and MCN-COOH, as well as loaded with B nanoparticles (MCN/B, MCN-COON/B), was studied by low-temperature nitrogen adsorption in a Quantachrome NOVA 1200e (Boynton Beach, FL, USA) Instrument. Specific surface areas were determined from the Brunauer Emmett Teller (BET) equation, and total pore volumes, mean diameters, and pore size distributions were calculated using the NLDFT equilibrium model. 

### 3.10. Transmission Electron Microscopy (TEM)

The size and the porous structure of the non-carboxylated and carboxylated empty and loaded MCN were observed using transmission electron microscopy (JEOL JEM 2100 h STEM, Tokyo, Japan) operating with 200 kV and point resolution = 0.23 nm. Samples were prepared by inserting an ethanol dispersion of the nanoparticles on a polymer microgrid supported on a Cu grid. The solvent was further evaporated under vacuum. All of the samples were investigated under 40 k and 100 k magnifications.

### 3.11. In Vitro Drug Release Study

An incubator shaker (Julabo Shake Temp SW23, Seelbach, Baden-Württemberg, Germany) was used for the present in vitro drug release studies. Along with 5 mg of free bicalutamide, drug-loaded particles (equivalent to 5 mg bicalutamide) were placed into either 50 mL hydrochloric acid buffer (pH 1.2), Sorensen phosphate buffer (pH 6.8), or citrate buffer (pH 5.0) containing 10% DMSO and 1% SLS, which ensured maintaining sink conditions. The temperature was kept at 37 °C under permanent stirring (100 rpm). Samples (2 mL) were withdrawn at appropriate time intervals as particles were removed from solution by centrifugation at 15,000 rpm for 2 min (High-Speed Mini Centrifuge Dragon Lab D2012 plus, Beijing, China). The experiments were performed in triplicate with or without NIR-irradiation for 3 min (808 nm NIR laser, 3 W/cm^2^ density). Irradiation was performed immediately after sampling, just before centrifugation. The concentration of the released bicalutamide was determined by UV–vis spectrophotometry at a wavelength of 270 nm. In order to maintain sink conditions, the 2 mL samples were returned to the media after the spectrophotometric determination. The standard curves (r^2^ > 0.995) were prepared in the concentration range of 5–100 μg/mL in all three different media. The cumulative drug release, as well as the time for 50% of released API (T_50_), were used to characterize the drug release.

### 3.12. Release Kinetics Study

The average cumulative release data of the three experiments taken from the in vitro release study of the prepared formulations in medium with pH 5.0 were used to determine the release kinetics of bicalutamide. The theoretical models describing zero-order, first-order, Higuchi, and Korsemeyer–Peppas kinetics were determined via the standard kinetics equation. The best fit model was determined according to the closeness of the value of the correlation coefficient (R^2^) to 1.

### 3.13. In Vitro Cell Viability Study

#### 3.13.1. Cell Culture and Treatment

LNCaP (androgen-sensitive human prostate adenocarcinoma cells) were obtained from the European Collection of Cell Cultures (ECACC, Porton Down, Salisbury, Wiltshire, UK). Cells were cultured in RPMI, supplemented with L-glutamine and fetal bovine serum (FBS) in an incubator, maintaining a constant 37 °C, 5% CO_2_, and maximum humidity. The preparation of the experimental setup included pipetting cell suspension in 96-well plates at 2 × 10^4^ cells/well. Cells were allowed to attach for 24 h, after which they were treated with either bicalutamide (12.5, 50.0, 100.0, and 200.0 µM) or bicalutamide-loaded MCN/B or MCN-COOH/B (bicalutamide in equimolar concentrations). In order to evaluate the effects of the empty drug-delivery systems, LNCaP cells were also treated with the non-loaded MCN and MCN-COOH. Plates were assigned to two identical groups, and, immediately after treatment, plates of the first group were irradiated with NIR light (808 nm NIR laser, 3 W/cm^2^ density) for 5 min at a 20 cm distance in order to study the presence of potential photothermal effects, and the other group was not irradiated (adapted from [3]). Cell viability of the cells in all the plates was evaluated after 24 h, 48 h, and 72 h. 

#### 3.13.2. Cytotoxicity Assay

The contents of the 96-well plates were aspirated and exchanged with a solution of MTT in culture media (final concentration of 5 mg/mL) and incubated for 3 h. Afterwards, the contents of all wells were exchanged with DMSO in order to dissolve the resulting formazan crystals in live cells. After an additional 30 min of incubation in a place protected from light, the absorbances in all wells at 570 nm and the reference wavelength of 960 nm were evaluated in a plate reader (Synergy 2, BioTek Instruments, Inc., Highland Park, Winooski, VT, USA). The viability values were expressed as normalized percent values versus the averaged values obtained from the control group.

#### 3.13.3. Statistical Analyses

All statistical analyses were performed on GraphPad Prism software (version 6, GraphPad Software, La Jolla, CA, USA). The dose-response relationships for every treatment group were modelled by non-linear regression, and IC50 values were obtained. Cells treated with different concentrations of MCN/B and MCN-COOH/B were compared via multiple *t*-tests with Holm-Sidak’s correction for multiple testing. The same method was applied for comparing cells, respectively, irradiated or not with NIR.

## 4. Conclusions

An effective anticancer drug, bicalutamide, was physically loaded into non-carboxylated and carboxylated mesoporous carbon nanoparticles with high encapsulation efficiency. The obtained systems, MCN/B and MCN-COOH/B, possessed a spherical shape, suitable particle size, NIR-triggered release of API, as well as good cytotoxic potential on LNCaP prostate cancer cells. However, the internal and surface hydrophobicity of the non-modified MCN/B nanoparticles resulted in poor water dispersibility, which limited their applicability due to aggregation and premature release of bicalutamide in aqueous media. Modification of the MCN surface with carboxyl groups improved the loading capacity and dispersion stability of the system as well as sustained bicalutamide release rate (zero-order release kinetics was observed with reduced burst effect), since the surface functional groups of the carrier were able to form hydrogen bonds with the API. Moreover, MCN-COOH/B exhibited improved cytotoxicity, compared to free bicalutamide and MCN/B, with a more pronounced difference between irradiated and non-irradiated groups. The increased cytotoxic potential of MCN-COOH/B, especially after NIR light irradiation, could reduce the therapeutic dose of bicalutamide, minimizing side effects, resulting in higher chemotherapeutic efficacy. Considering all results, we believe that the obtained carboxylated mesoporous carbon nanoparticles could be considered as potential drug delivery systems for combined chemo-photothermal therapy of prostate cancer.

## Figures and Tables

**Table 1:** Encapsulation efficiency (EE%) and loading capacity (LC%) of MCN/B and MCN-COOH/B, mean ± SD, n = 3.

Parameter	Sample Coding	
	MCN/B	MCN-COOH/B
EE%	84.15 ± 4.3	99.35 ± 3.1
LC%	41.08 ± 2.5	48.53 ± 1.8

**Table 2:** FTIR spectra (characteristic peaks) of MCN, MCN-COOH, bicalutamide (B), MCN/B, and MCN-COOH/B: υ—stretching vibrations; β—bending vibrations.

Functional Group Vibration	Sample Coding				
	MCN	MCN-COOH	B	MCN/B	MCN-COOH/B
υ O-H /hydroxyl group/			3597 cm^−1^	3595 cm^−1^	3579 cm^−1^
υ N-H /amide group/			3336 cm^−1^	3334 cm^−1^	3327 cm^−1^
υ C–H /aromatic/			3070 cm^−1^	3070 cm^−1^	3070 cm^−1^
υ C–H /aliphatic/			2975 cm^−1^	2975 cm^−1^	2975 cm^−1^
υ C≡N /nitrile group/			2231 cm^−1^	2231 cm^−1^	2231 cm^−1^
υ C=O /carbonyl group/		1707 cm^−1^	1696 cm^−1^	1705 cm^−1^	1684 cm^−1^
υ C=C /aromatic/	1597 cm^−1^	1598 cm^−1^	1602 cm^−1^	1579 cm^−1^	1581 cm^−1^
β C-H /aromatic/			1514 cm^−1^	1514 cm^−1^	1514 cm^−1^
υ S=O /sulfonyl group/			1327 cm^−1^	1327 cm^−1^	1325 cm^−1^
υ C–O		1256 cm^−1^	1258 cm^−1^	1257 cm^−1^	1253 cm^−1^
υ C–F /monofluorinated benzene/			1231 cm^−1^	1230 cm^−1^	1228 cm^−1^
υ C–F /CF3/			1126 cm^−1^	1126 cm^−1^	1126 cm^−1^

**Table 3:** Particle size, PDI, and Z-potential of the empty and bicalutamide-loaded MCN and MCN-COOH nanoparticles in different media; mean ± SD; n = 3.

Parameter		Sample Coding			
	Media	MCN	MCN-COOH	MCN/B	MCN-COOH/B
Size, nm	Deionized water	103.2 ± 5.8	174.8 ± 2.4	125.7 ± 6.1	198.2 ± 3.2
	Buffer pH 1.2	120.4 ± 6.3	222.7 ± 6.5	137.4 ± 7.1	248.1 ± 6.4
	Buffer pH 5.0	109.7 ± 4.1	203.1 ± 5.9	131.7 ± 6.5	210.8 ± 5.7
	Buffer pH 6.8	105.4 ± 5.9	181.7 ± 3.2	129.1 ± 5.7	195.9 ± 6.2
PDI	Deionized water	0.451	0.157	0.567	0.225
	Buffer pH 1.2	0.625	0.287	0.739	0.301
	Buffer pH 5.0	0.542	0.204	0.617	0.265
	Buffer pH 6.8	0.448	0.149	0.530	0.238
Z-potential, mV	Deionized water	−9.02 ± 0.3	−38.05 ± 0.4	−7.03 ± 0.2	−34.13 ± 0.6
	Buffer pH 1.2	−8.12 ± 0.6	−30.25 ± 0.6	−6.12 ± 0.3	−29.78 ± 0.2
	Buffer pH 5.0	−8.51 ± 0.5	−34.48 ± 0.5	−6.53 ± 0.4	−31.74 ± 0.5
	Buffer pH 6.8	−8.84 ± 0.3	−39.11 ± 0.6	−7.18 ± 0.2	−33.65 ± 0.7

**Table 4:** Texture parameters of free bicalutamide, empty, and bicalutamide-loaded MCN and MCN-COOH nanoparticles.

Sample Coding	Parameter					
	SBET, m^2^/g	Vt, cm^3^/g	Dav, nm	Vmi, cm^3^/g	Smi, m^2^/g	Sext, m^2^/g
B	11	0.02	6.0	-	-	11
MCN	246	0.49	7.9	0.02	49	197
MCN/B	54	0.29	21.8	-	-	54
MCN-COOH	16	0.06	14.5	-	-	16
MCN-COOH/B	17	0.09	21.0	-	-	17

**Table 5:** Time for 50% bicalutamide release (T_50_) from free bicalutamide (B) and loaded nanoparticle formulations MCN/B and MCN-COOH/B in different pH media with or without NIR light; mean ± SD; n = 3.

Sample Coding	T_50_ of Bicalutamide, h		
	pH 1.2	pH 5.0	pH 6.8
B	0.49 ± 0.05	0.45 ± 0.02	0.42 ± 0.01
B + NIR	0.47 ± 0.03	0.46 ± 0.01	0.44 ± 0.03
MCN/B	3.85 ± 0.31	3.71 ± 0.21	3.68 ± 0.34
MCN/B + NIR	3.03 ± 0.26	2.94 ± 0.36	2.79 ± 0.21
MCN-COOH/B	12.09 ± 0.43	10.87 ± 0.57	10.15 ± 0.71
MCN-COOH/B + NIR	7.96 ± 0.57	6.76 ± 0.49	6.03 ± 0.35

**Table 6:** Kinetic model fitting results for bicalutamide release from nanoparticle formulations in citrate buffer medium with pH 5.0.

Sample Coding	Kinetics Model							
	Zero-Order		First-Order Kinetics		Higuchi Model		Korsmeyer–Peppas Model	
	Qt = Q0 + k0t		lnQt = lnQ0 − k1t		Qt = kHt1/2		Qt/Q∞ = kPtn	
	R^2^	k	R^2^	k	R^2^	k	R^2^	n
MCN/B	0.9515	0.0015	0.6145	0.0051	0.9959	3.8098	0.6800	−0.4342
MCN/B + NIR	0.9385	0.0016	0.5739	0.0050	0.9980	3.9936	0.6815	−0.4458
MCN-COOH/B	0.9673	0.0009	0.9224	0.0075	0.8224	2.0711	0.5638	−0.8572
MCN-COOH/B + NIR	0.9967	0.0012	0.8386	0.0069	0.9298	2.9128	0.5033	−1.0811

## Data Availability

Data are available from the authors (see given emails).

## Supplementary Materials

The following supporting information can be downloaded at: https://www.mdpi.com/article/10.3390/molecules30153055/s1, Figure S1. FTIR spectra of MCN, MCN-COOH, bicalutamide (BLT), MCN/B and MCN-COOH/B. Figure S2. Particle size (a), PDI (b), Z-potential (c), and % turbidity (d) of MCN and MCN-COOH nanoparticles, measured at different time points, mean ± SD, n = 3.

## Abbreviations

The following abbreviations are used in this manuscript:

API	Active Pharmaceutical Ingredient
ATR-FTIR	Fourier-Transform Attenuated Total Reflection Infrared
B	Bicalutamide
BCS	Biopharmaceutic Classification System
BET	Brunauer Emmett Teller
DLS	Dynamic Light Scattering
DMSO	Dimethylsulfoxide
EDTA	Ethylenediaminetetraacetic Acid
EE	Encapsulation efficiency
FBS	Fetal Bovine Serum
LC	Loading capacity
LNCaP	Androgen-Sensitive Human Prostate Adenocarcinoma Cells
MCN	Mesoporous Carbon Nanoparticles
NIR	Near-Infrared
PDI	Polydispersity Index
PEG	Polyethylene Glycol
PTT	Photothermal Therapy
PVP	Polyvinylpyrrolidone
SLS	Sodium Lauryl Sulphate
TEM	Transmission Electron Microscopy
XRD	X-ray Diffraction
Z-potential	Zeta-potential
