# Supplementary material for: Carboxylated Mesoporous Carbon Nanoparticles as Bicalutamide Carriers with Improved Biopharmaceutical and Chemo-Photothermal Characteristics
Source: Molecules. 2025 Jul 22;30(15):3055. doi: 10.3390/molecules30153055 (PMC12348955; doi:10.3390/molecules30153055)
Supplement: Supplementary file 1 [file molecules-30-03055-s001.zip › molecules-3739020-supplementary.pdf]

Supplementary material

Molecules

Carboxylated Mesoporous Carbon Nanoparticles as Bicalutamide Carriers with Improved Biopharmaceutical and Chemo-Photothermal Characteristics

Teodora Popova [1,*], Borislav Tzankov [1], Marta Slavkova [1], Yordan Yordanov [2], Denitsa Stefavova [2], Virginia Tzankova [2], Diana Tzankova [3], Ivanka Spasova [4], Daniela Kovacheva [4], Christina Voycheva [1,*]

[1] Department of Pharmaceutical Technology and Biopharmaceutics, Faculty of Pharmacy, Medical University of Sofia, Bulgaria; btzankov@pharmfac.mu-sofia.bg (B.T.); mslavkova@pharmfac.mu-sofia.bg (M.S.)
[2] Department of Pharmacology, Pharmacotherapy and Toxicology, Faculty of Pharmacy, Medical University of Sofia, Bulgaria; yyordanov@pharmfac.mu-sofia.bg (Y.Y.); denitsa.stefanova@pharmfac.mu-sofia.bg (D.S.); vtzankova@pharmfac.mu-sofia.bg (V.T.)
[3] Department of Pharmaceutical Chemistry, Faculty of Pharmacy, Medical University of Sofia, Bulgaria; d.tsankova@pharmfac.mu-sofia.bg (D.T.)
[4] Institute of General and Inorganic Chemistry, Bulgarian Academy of Sciences, Sofia, Bulgaria; ispasova@svr.igic.bas.bg (I.S.), didka@svr.igic.bas.bg (D.K.)
* Correspondence: tpopova@pharmfac.mu-sofia.bg (T.P.); hvoycheva@pharmfac.mu-sofia.bg (C.V.)

1. Fourier-transform attenuated total reflection infrared (ATR-FTIR) spectroscopy

The FTIR spectra of the prepared empty and bicalutamide loaded MCN and MCN-COOH are presented in Figure S1.

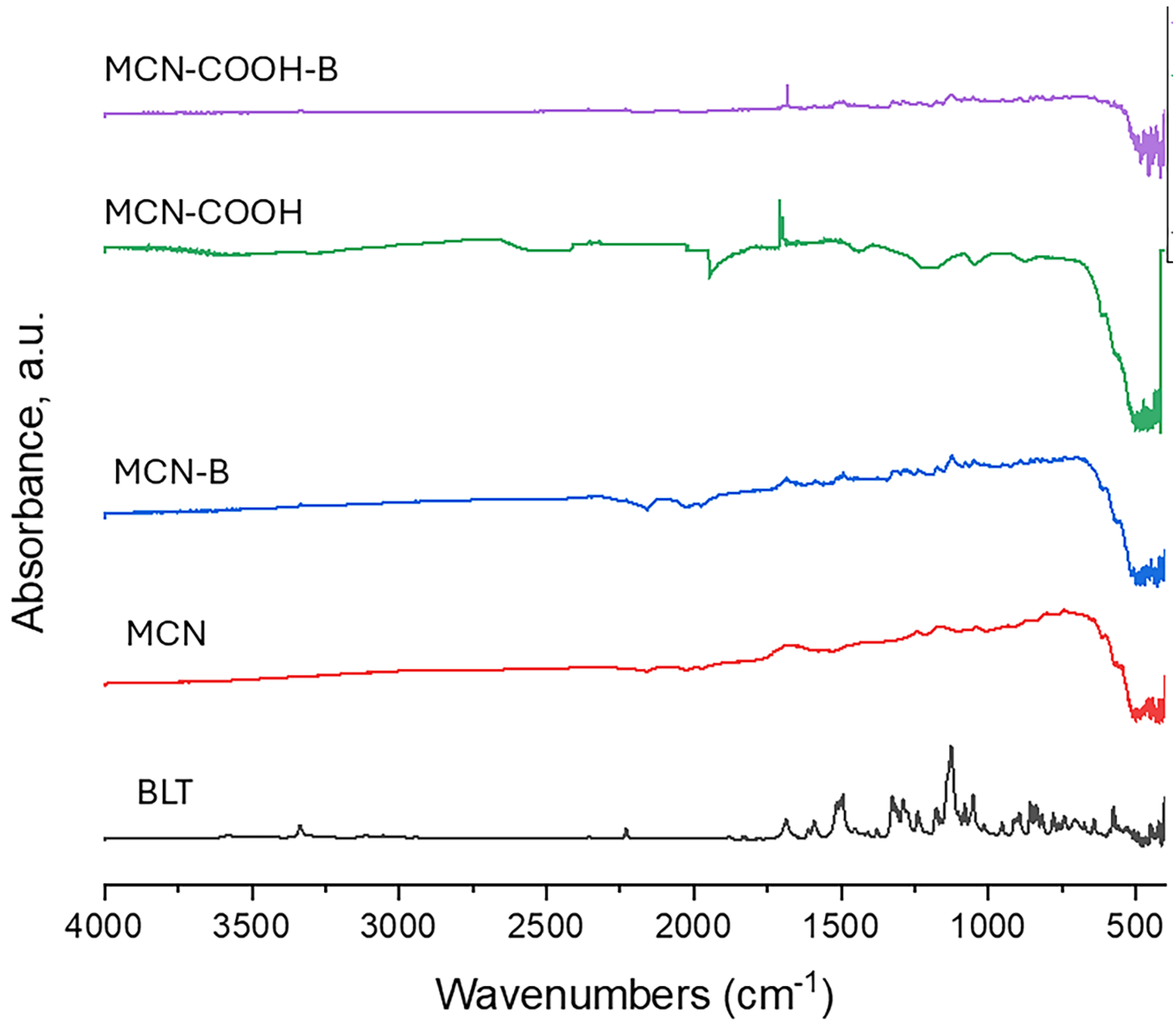

Figure S1. FTIR spectra of MCN, MCN-COOH, bicalutamide (BLT), MCN/B and MCN-COOH/B.

2. Dispersion stability study of MCN and MCN-COOH

Figure S1 contains the data, obtained from the performed DLS and turbidity measurement.

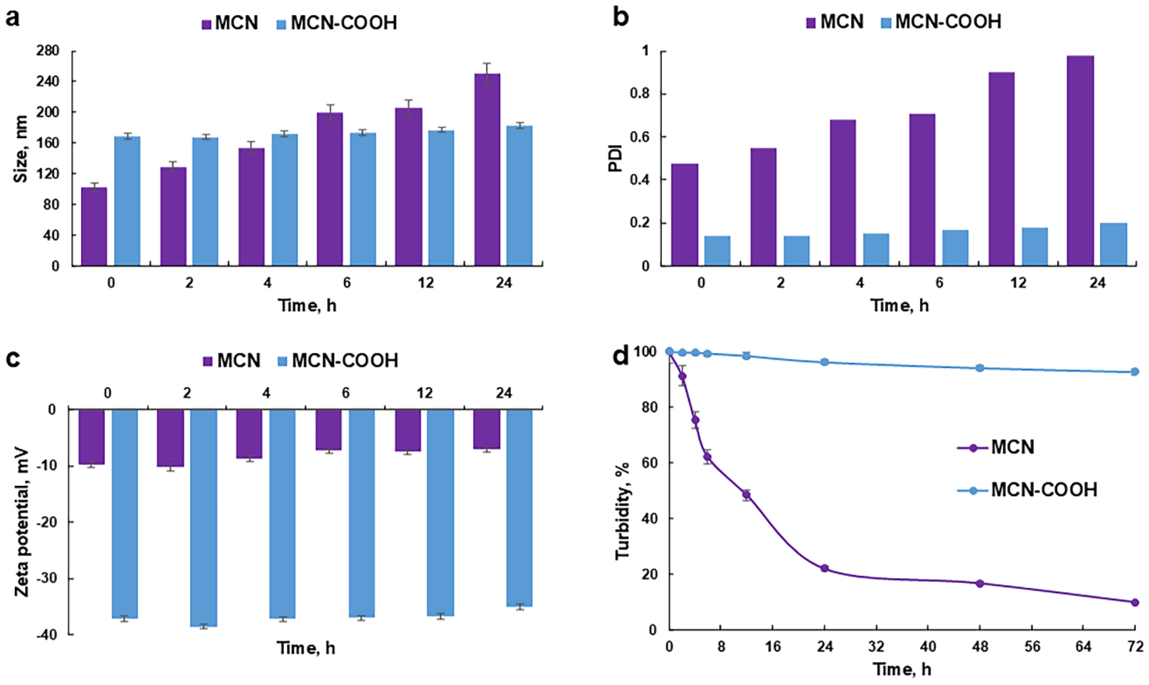

Figure S2. Particle size (a), PDI (b), Z-potential (c) and % turbidity (d) of MCN and MCN-COOH nanoparticles, measured at different time points, mean ± SD, n=3.
